# Exploring the Antioxidant Potential of *Talisia esculenta* Using In Vitro and In Vivo Approaches

**DOI:** 10.3390/nu15173855

**Published:** 2023-09-04

**Authors:** Maria Lúcia da Silva Cordeiro, Verônica Giuliani de Queiroz Aquino-Martins, Ariana Pereira da Silva, Georggia Fatima Silva Naliato, Elielson Rodrigo Silveira, Raquel Cordeiro Theodoro, Deborah Yara Alves Cursino da Santos, Hugo Alexandre Oliveira Rocha, Katia Castanho Scortecci

**Affiliations:** 1 Laboratório de Transformação de Plantas e Análise em Microscopia (LTPAM), Departamento de Biologia Celular e Genética, Universidade Federal do Rio Grande do Norte (UFRN), Natal 59078-970, RN, Brazil; mlscordeiro04@gmail.com (M.L.d.S.C.); verinhaquino@hotmail.com (V.G.d.Q.A.-M.); arianapereirauf@gmail.com (A.P.d.S.); 2 Programa de Pós-graduação em Bioquímica e Biologia Molecular, Centro de Biociências, Universidade Federal do Rio Grande do Norte (UFRN), Natal 59078-970, RN, Brazil; georggianaliato@gmail.com (G.F.S.N.); raquel.ctheodoro@gmail.com (R.C.T.); hugo.rocha@ufrn.br (H.A.O.R.); 3 Instituto de Medicina Tropical, Universidade Federal do Rio Grande do Norte (UFRN), Natal 59077-080, RN, Brazil; 4 Laboratório de Fitoquímica, Departamento de Botânica, Universidade de São Paulo (USP), São Paulo 05508-090, SP, Brazil; elielson.bio@ib.usp.br (E.R.S.); dyacsan@ib.usp.br (D.Y.A.C.d.S.); 5 Laboratório de Biotecnologia de Polímeros Naturais (BIOPOL), Departamento de Bioquímica, Universidade Federal do Rio Grande do Norte (UFRN), Natal 59078-970, RN, Brazil

**Keywords:** medicinal plant, phenolic compounds, Sapindaceae, *Tenebrio molitor*, oxidative damage, CuSO_4_ stress

## Abstract

Medicinal plants, such as *Talisia esculenta*, are rich in antioxidant biomolecules, which are used in the treatment and prevention of many diseases. The antioxidant potential of *T. esculenta* extracts obtained from leaves and fruit peels was investigated using biochemical and 3T3 cell line assays as well as in vivo assays using an organism model *Tenebrio molitor*. Four extracts were tested: hydroethanolic extracts from leaves (HF) and from fruit peels (HC), and infusion extracts from leaves (IF) and from fruit peels (IC). The biochemical assays demonstrated an antioxidant capacity verified by TAC, reducing power, DPPH, and copper chelating assays. None of the extracts exhibited cytotoxicity against 3T3 cells, instead offering a protection against CuSO_4_-induced oxidative stress. The antioxidant activity observed in the extracts, including their role as free radical scavengers, copper chelators, and stress protectors, was further confirmed by *T. molitor* assays. The CLAE-DAD analysis detected phenolic compounds, including gallic acid, rutin, and quercitrin, as the main constituents of the samples. This study highlights that leaf and fruit peels extracts of *T. esculenta* could be effective protectors against ROS and copper-induced stress in cellular and invertebrate models, and they should be considered as coadjutants in the treatment and prevention of diseases related to oxidative stress and for the development of natural nutraceutical products.

## 1. Introduction

Organisms are exposed daily to oxidative stress either by cell metabolism or external factors such as food, chemical compounds, viruses, and bacteria. To reduce the negative effects of such stress, cells have different mechanisms to ensure an efficient endogenous antioxidant system comprising enzymatic and non-enzymatic components that counteracts ROS/RNS [1,2]. However, when this system fails during ROS/RNS elimination, a redox imbalance, known as oxidative or nitrosative stress, is created that may lead to different diseases such as cancers [3,4], inflammatory bowel disease [5], diabetes [6] cardiovascular disease [7], and Alzheimer’s disease [8], as well as neurological disorders such as depression and anxiety [9].

Plants are equipped with an impressive arsenal of bioactive molecules, known as secondary metabolites, that serve vital functions in both plant defense and adaptation to a wide range of environmental conditions [10]. Some of these molecules present an antioxidant activity that helps cells to reduce ROS/RNS. Thus, plenty of research has been conducted to investigate molecules that may be able to mitigate the harmful effects of reactive species and have a pharmacological activity against human pathologies [11,12]. Phenolic compounds such as polyphenols, flavonoids, phenolic acids, and anthocyanins have already shown immense potential as antioxidants and anti-inflammatory agents [13,14,15].

Numerous plant extracts have undergone thorough investigation, revealing their remarkable potential as natural antioxidant molecules due to their low toxicity and diverse composition, making them promising candidates for supplementation or medicinal purposes, offering an attractive alternative to synthetic counterparts [16,17]. Furthermore, certain bioactive molecules extracted from plants, such as taxol, vincristine, and vinblastine, have made significant contributions to cancer treatment. Additionally, notable examples include atropine, which exhibits an antispasmodic and antiparkinsonian activity, and codeine, which is widely used as an analgesic. These compounds underscore the invaluable role of plant-derived substances in the development of therapeutic agents [18,19]. These examples highlight the importance of investigating bioactive molecules as well as their pharmacological characterization.

*Talisia esculenta* (St. Hil.) Raldk, commonly known as “pitombeira”, is a native plant found in the Caatinga and Atlantic Forest biomes of Brazil [20,21]. Leaf infusions of *T. esculenta* have been traditionally employed in folk medicine as analgesics for back problems, rheumatism, and hypertension [22,23]. Fruits from *T. esculent* also hold significant value among local communities and have recently gained attention for their gourmet applications, highlighting their growing importance beyond traditional uses [24,25]. In various culinary practices, fruit peels are often discarded, disregarding their potential as a rich source of bioactive molecules. However, recent studies have highlighted that fruit peels can be an important source of polyphenols, making them a valuable resource with pharmacological and nutraceutical applications [26,27,28,29,30,31].

Despite the use of *T. esculenta* leaves in traditional medicine, there are still significant gaps in the knowledge about its pharmacological properties and underlying mechanisms. Based on these considerations, our hypothesis is that the extracts of the leaves and fruit peels of *T. esculenta* would be a source of bioactive compounds with an antioxidant potential both *in vitro* and *in vivo*. In this study, we investigated the antioxidant capacity of the infusion and the hydroethanolic of the leaves and fruit peel extracts of *T. esculenta.* We used *in vitro* biochemical assays and included the NHI/3T3 cell line. Additionally, an *in vivo* assay was conducted using the *Tenebrio molitor* animal model. Furthermore, a phytochemical analysis was conducted to identify the potential bioactive molecules present in these extracts. The data presented here contribute to the understanding of the bioactive potential and mechanisms of action associated with *T. esculenta* extracts.

The extracts obtained from *T. esculenta* demonstrated the ability to neutralize oxidative damage caused by reactive oxygen species (ROS) and copper-induced stress, which may have significant implications for the treatment of oxidative diseases. Thus, considering its phytochemical composition, which is rich in polyphenols and is associated with its bioactivity, it can be inferred that *T. esculenta* is a medicinal source of bioactive compounds with potential applications for promoting human health and obtaining pharmaceutical and food products.

## 2. Materials and Methods

### 2.1. Materials

Ethylenediaminetetraacetic acid (EDTA), gallic acid, ascorbic acid, quercetin, methionine, pyrocatechol violet, riboflavin, and ammonium molybdate were obtained from Sigma-Aldrich Co. (St. Louis, MO, USA). Potassium ferricyanide, trichloroacetic acid, Folin-Ciocalteu reagent, and sulfuric acid were purchased from Merck (Darmstadt, Germany). DPPH (2,2-diphenyl-1-picrylhydrazyl) was purchased from Fluka (Seelze, Germany). Cupric sulfate was obtained from Chemical Kinetics. Dulbecco’s modified Eagle’s medium (DMEM) and fetal bovine serum (FBS) were obtained from CULTILAB (Campinas, SP, Brazil). Penicillin and streptomycin were obtained from Gibco (Fort Worth, TX, USA). All of the other reagents were of analytical grade.

### 2.2. Plant Material

The leaves and fruits of *T. esculenta* were harvested in the city of Parnamirim/RN, in March 2020, under a license from the Biodiversity Authorization and Information System-SISBIO with registration number 70956 and SISGEN A984A1D. A voucher specimen was deposited in the herbarium of the Federal University of Rio Grande do Norte-UFRN, with the registration UFRN 26618. The botanical identification was completed by Dr. Leonardo de Melo Versieux (UFRN).

### 2.3. Preparation of Extracts

In this work, we chose to focus on leaves and fruit peels, using two different extraction methods: infusion and hydroethanolic extraction. The infusion was selected to align with the practices commonly employed by folk medicine when preparing extracts and to give scientific data for its use. This aqueous preparation is also a popular low-cost therapeutic alternative capable of extracting antioxidant compounds with protective effects against diseases [32,33]. Maceration using a mixture of ethanol and water was employed because this is a simple and efficient technique for extracting phenolic compounds from plant samples, and ethanol is a low-toxicity organic solvent [34,35]. The leaves and peels were washed with running water to remove residues and they were then disinfected with 1% sodium hypochlorite for 5 min. After this time, the plant material was rinsed six to eight times with distilled water. The leaves and fruit peels were triturated, and a solvent was added for the extraction process.

An aqueous extract (infusion) using leaves was prepared according to [36] with modifications. Briefly, 1000 mL of boiling distilled water (100 °C) was added to 50 g of plant material, which was incubated in the dark at room temperature for 30 min. After this period, the infusion was filtered through Whatman No. 1, the solid residue was discarded, and the infusion volume was transfer to amber bottle and the volume was reduced using a lyophyllizer (Freezone 4.5, Labconco, Kansas City, MI, USA). For hydroethanolic extract preparation, 100 g of plant material (leaves or fruit peels) was macerated with 1000 mL of 70% ethanol (1:10 ratio). The extract was kept at room temperature under 150 rpm rotation, and it was protected from light for 24 h [37]. The hydroethanolic extract was filtered using Whatman No. 1, and the solid residue was discarded. The organic solvent was removed from the extract using a rotary evaporator (TE210, Tecnal, Piracicaba, Brazil) at 40 °C. The extracts were named as follows: hydroethanolic extract of leaves (HF), infusion of leaves (IF), hydroethanolic extract of fruit peels (HC), and infusion of fruit peels (IC).

### 2.4. Antioxidant Capacity In Vitro

The antioxidant potential from the samples was tested by their ability to act as a free radical-reducing agent or a metal chelator. The following assays were completed: total antioxidant capacity (TAC), DPPH radical scavenging, reducing power, and copper chelation as described by [38,39].

#### 2.4.1. Total Antioxidant Capacity (TAC)

This test measures the ability of the compounds present in the extracts to act as reducing agents and it is based on the reduction of Molybdenum^+6^ to Molybdenum^+5^, with the formation of a green-colored complex (phosphate/Mo^+5^) at an acidic pH. For this, a tube was prepared with ammonium molybdate (4 mM), sulfuric acid (0.6 M), sodium phosphate (28 mM), and the extracts (10 mg/mL) into final volume of 1 mL. The tubes were mixed and incubated in an incubator at 100 °C for 90 min; after this period, the samples were measured by reading the absorbance at 695 nm at spectrophotometer (Biotek Epoch Microplate, Santa Clara, CA, USA). The antioxidant capacity (TAC) was expressed as the ascorbic acid equivalent per mg of the sample.

#### 2.4.2. Reducing Power

Extracts at different concentrations (50 µg/mL, 100 µg/mL, or 250 µg/mL) were incubated with a solution containing phosphate buffer (0.2 M, pH 6.6) and potassium ferricyanide (1%) at 50 °C for 20 min, into a final volume of 4 mL. The reaction was terminated by adding trichloroacetic acid (10%) followed by homogenization with distilled water and ferric chloride (0.1%). The absorbance was measured at 700 nm using a spectrophotometer (Biotek Epoch Microplate, Santa Clara, CA, USA). The percentage of reducing capacity of the samples was calculated, considering as 100% activity of the reducing capacity of ascorbic acid at 0.1 mg/mL.

#### 2.4.3. DPPH Radical Scavenging

The test assesses the ability of the compounds present in the extracts to act as hydrogen donors, reducing the DPPH free radical and eliminating it from the solution. In a 96-well plate, extracts at different concentrations (50 µg/mL, 100 µg/mL, or 250 µg/mL) were added, followed by the DPPH solution. The control was the reaction only with DPPH. The mixture was incubated in the dark at room temperature for 30 min. The absorbance was measured at 517 nm using a spectrophotometer (Biotek Epoch Microplate, Santa Clara, CA, USA), and the results were expressed as percent scavenging obtained by the following formula:

$$\%\;\mathrm{Scavenging}=\left[1-\left(\frac{A.\;\mathrm{Sample}}{A.\;\mathrm{Control}}\times 100\right)\right]$$

where the control is DPPH solution

#### 2.4.4. Copper Chelation

The copper chelating capacity of the extracts was measured using the pyrocatechol violet reagent, which, in the presence of chelating compounds, does not associate with copper and does not form a colored complex, resulting in a decrease in the color of the solution. For this assay, extracts at different concentrations (50 µg/mL, 100 µg/mL, or 250 µg/mL), pyrocatechol violet (4 mM), and copper sulfate pentahydrate (50 μg/mL in acetate buffer) were added to 96-well microplates into a final volume of 336 µL. The mixture was homogenized, and the chelation capacity of the copper ions was measured by reading the absorbance at 632 nm using a spectrophotometer (Biotek Epoch Microplate, Santa Clara, CA, USA).

### 2.5. Cell Line Assays

#### 2.5.1. MTT Reduction Assay

NIH/3T3 cells (ATCC CRL-1658) were grown in 96-well plates at a density of 10 × 10^4^ cells/well in DMEM medium supplemented with 10% fetal bovine serum (FBS) and it was incubated at 37 °C and 5% CO_2_. After 24 h, the medium was replaced, and cells were incubated with DMEM medium without FBS. After another 24 h, the medium was removed, and a new DMEM medium with 10% FBS with different concentrations of extracts (50 µg/mL, 100 µg/mL, or 250 µg/mL) was added to the wells. After 24 h of incubation, the MTT assay was permormed according to [40] to analyze the effect of the extracts on cells. After conducting this analysis, the medium was removed and the MTT reagent solution (1 mg/mL) was added to the wells and incubated for 4 h at 37 °C and 5% CO_2_. After 4 h of incubation, the formazan crystals that formed were solubilized by adding absolute ethanol P.A., and the absorbance was measured at 570 nm using a microplate reader (Biotek Epoch Microplate, Santa Clara, CA, USA). The negative control comprised cells grown only in DMEM medium with FBS without the presence of extracts. This absorbance was considered as 100% MTT reduction. The percentage of MTT reduction by cells treated with the extracts was calculated using the following equation:

$$\;\mathrm{MTT}\;\mathrm{reduction}\;\left(\%\right)=\frac{\mathrm{Sample}\;\mathrm{abs}\;\left(570\;\mathrm{nm}\right)}{\mathrm{Negative}\;\mathrm{Control}\;\mathrm{Abs}\;\left(570\;\mathrm{nm}\right)}\times 100$$

#### 2.5.2. Migration Assay

The evaluation of the influence of *T. esculenta* extracts on the migratory behavior of NIH/3T3 cells (ATCC CRL-1658) was conducted using a wound healing assay that was performed according to [41]. The cells were plated at a density of 1 × 10^6^ cells/well in 24-well plates in DMEM medium supplemented with 10% FBS. After 24 h, when a confluent monolayer was formed, the medium was removed, and the wells were washed with 500 µL PBS (sodium chloride 145 mM (0.85%) in 150 mM of phosphate buffer). A scratch was created in the cell monolayer using a 200 µL pipette tip, and the wells were washed again with PBS to remove cell debris. DMEM supplemented with 10% FBS with extracts at a concentration of 100 µg/mL was added to the wells. The wells were photographed at 0, 12, and 24 h after incubation using a Mioticam 5+ camera attached to a Nikon Eclipse Ti microscope with a 40× objective. Wound width measurements were performed using NIS-elements AR software by measuring the closest points on both sides of the wound [42]. The percentage of wound healing was calculated according to [41] using the following equation:

$$\;\mathrm{Wound}\;\mathrm{closure}\;\left(\%\right)=\frac{w0-wn}{w0}\times 100$$

where *wn* is the wound width after different time intervals and *w*0 is the initial width right after the scratch formation.

#### 2.5.3. In Vivo Oxidative Stress Assay Using CuSO_4_ and Ascorbate-Induced in a NIH/3T3 Cell Line

The antioxidant capacity of the extracts was also evaluated *in vivo* using a cell model. Copper sulfate (CuSO_4_) was used to induce oxidative stress in the cells, and the extracts’ capacity for protection was evaluated. This assay was performed as described by [43], with some modifications. First, the CuSO_4_ concentration was used to consider a stress condition. We used CuSO_4_ concentrations ranging from 1 μM to 30 μM and 1 mM ascorbate. The concentration of 25 μM CuSO_4_ was chosen as a 50% MTT reduction capacity was observed for NIH/3T3 cells. After that, the cells were plated into 96-well plates at a density of 10 × 10^4^ cells/well in DMEM medium containing 10% FBS at 37 °C and 5% CO_2_. After 24 h of incubation, a new DMEM medium was added without FBS for 24 h. Then, a new DMEM medium was added with extracts at 100 μg/mL, as well as 25 μM CuSO_4_. After 15 min, 1 mM ascorbate was added to the wells. The plates were incubated for 45 min at 37 °C and 5% CO_2_. After treatment, the medium was removed and a new DMEM medium with FBS was added, the cells were maintained for 24 h in incubation at 37 °C and 5% CO_2._ After this period the MTT assay was performed to evaluate the protection capacity of the extracts. The negative control comprised only DMEM medium with 25 μM of CuSO_4_ without ascorbate, and the positive control (for stress condition) comprised DMEM medium, 25 μM CuSO_4_, and 1mM ascorbate. The reading was performed using a microplate reader (Biotek Epoch Microplate, Santa Clara, CA, USA).

### 2.6. Antioxidant Capacity In Vivo Using Tenebrio molitor as an Animal Model

#### 2.6.1. *Tenebrio molitor* Maintenance

*Tenebrio molitor* were kept in plastic containers (30 × 45 × 15 cm) at room temperature (25 °C–29 °C) under darkness. The life-cycle stage (larvae, pulp, and beetle) was separated to optimize the colony development. The larval feeding substrate consisted of 36.36% wheat bran, 18.18% wheat germ, 18.18% wheat flour, 16.36% oat flakes, 10.9% soybean extract, and 0.04% chloramphenicol (Patricia Canteri de Souza, personal communication).

#### 2.6.2. Effect of the Extract on *T. molitor* Survival

Considering the results obtained with the 3T3 cell line two extracts were chosen for this animal model: leaf infusion (IF) and fruit peels of hydroethanolic extract (HC). First, we examined whether these extracts could have a toxicity effect. The animals (weighing 100/150 mg) were randomly divided into groups of 10 animals, and the assays were performed in biological triplicates, resulting in 30 animals per treatment. The animals were transferred to Petri dishes containing the substrate and were kept at room temperature. The animals were anesthetized using ice, and the IF and HC extracts (5 μL) were inoculated at a final concentration of 100 µg/mL and 250 µg/mL using a Hamilton Syringe (701 N, 26’s gauge, 10 μL). Inoculation was performed at the ventral portion of the animal’s hemocoel, between the second and third abdominal segments. For the negative control, the larvae were inoculated with PBS (137 mM NaCl, 2.7 mM KCl, 10 mM Na_2_HPO_4_, 1.76 mM KH_2_PO_4_, pH 7.4). Larval survival was evaluated for 10 days, with measurements taken every 24 h. Death was determined by the absence of movement of larvae in response to a mechanical stimulus, followed by the presence of melanization in the animal’s cuticle.

#### 2.6.3. Effect of IF and HC on *Tenebrio molitor* Survival after CuSO_4_ Induced Oxidative Stress

The assay was performed according to [44], with modifications. *T. molitor* larvae (weighing 100 mg to 150 mg) were selected randomly and divided into groups formed by 10 larvae, this assay was performed in biological triplicates. Consequently, 30 animals per treatment were used. These groups were transferred to Petri dishes and the larvae were anesthetized with ice and inoculated with 5 µL of 0.25% CuSO_4_ to induced oxidative stress. The inoculation was performed in the ventral portion of the animal’s hemocoel, between the second and third abdominal segments. After 1 h, 5 µL of the sample (IF and HC) was inoculated into the animals at a concentration of 250 µg/mL. The positive control was the larvae inoculated with 5 µL of 0.25% CuSO_4_, and after 1 h they were inoculated with 5 µL of PBS. The negative control was the larvae inoculated twice with 5 µL of PBS. The survival of the animals was evaluated for 15 days; the animals were checked every 24 h. The animals were considered dead in the absence of movement in response to a mechanical stimulus, accompanied by melanization of the cuticle.

#### 2.6.4. Analysis of Melanization after CuSO_4_ Oxidative Induced Stress

The quantification of melanization was measured according to [45], with modifications. For each group, five larvae were used, resulting in a total of 15 larvae per treatment because it three biological replicates were performed. The animals used in the oxidative stress induction experiment with CuSO_4_ were anesthetized on ice; with the assistance of a scalpel blade, an incision was made in the head region to collect the internal contents of the animal. The collected material was homogenized with PBS at a ratio of 1:100 (larvae/μL volume) into 1.5 mL conic tubes. Subsequently, the solution was centrifuged at 21,912.8× *g* for 15 min at 4 °C. The supernatant was used for the quantification of melanization, which was determined by measuring the absorbance at 405 nm using a microplate reader (Biotek Epoch Microplate, Santa Clara, CA, USA).

### 2.7. Chemical Constituents of T. esculenta Extracts

#### 2.7.1. Quantification of Total Phenolic Compound Content

The total contents of the phenolic compounds were determined with a colorimetric method using the Folin−Ciocalteu reagent (Sigma-Aldrich), following the protocol described by [46]. Gallic acid was used as the reference standard for the calibration curve, and the results were expressed as gallic acid equivalents/mg of extract.

#### 2.7.2. Quantification of the Total Flavonoid Content

The concentration of flavonoids was determined using the Aluminum Chloride (AlCl_3_) method described by [47], with modifications. A solution containing the samples (10 mg/mL), aluminum chloride (1.8%), and sodium acetate (8.2%) was prepared. The mixture was then incubated for 40 min in the dark, and the absorbance was measured at 415 nm using a spectrophotometer (Biotek Epoch Microplate, Santa Clara, CA, USA). A standard curve was constructed using quercetin (concentration ranging from 1.25 to 75 μg/mL). The results were expressed as quercetin equivalents/mg extract.

#### 2.7.3. High-Performance Liquid Chromatography with Diode Array Detection (CLAE-DAD)

CLAE-DAD analysis was performed as described by [48,49]. An Agilent 1260 system equipped with a Zorbax C18 column (150 mm × 4.6 mm × 3.5 µm) at 45 °C was used. A gradient of 0.1% acetic acid (AcOH) and acetonitrile (CH_3_CN) was employed as the mobile phase, and the start composition was 10% CH_3_CN (0–6 min), followed by an increase to 15% (6–7 min), remaining isocratic for 15 min, followed by an increase to 50% (22–32 min) and to 100% (32–42 min), and then kept in isocratic conditions for an additional 8 min. The solvent flow rate was set at 1.0 mL/min, and the injection volume for analysis was 10 µL at a concentration of 2 mg/mL. The chromatogram was processed with λ = 352 nm, and compound suggestions were based on the analysis of absorption-UV−visible spectra and through a comparison of retention times with standards.

### 2.8. Statistical Analysis

All of the experiments were performed in triplicate, and the data were expressed as mean ± standard deviation. The values were subjected to analysis of variance (ANOVA), and the means were compared using Tukey and Dunnett tests (*p* < 0.05), with statistical analysis performed using GraphPad Prism 9 software (GraphPad Software, San Diego, CA, USA). Two or three independent experiments were conducted.

## 3. Results

### 3.1. In Vitro Antioxidant Activity of TE Extracts

**Figure 1:**
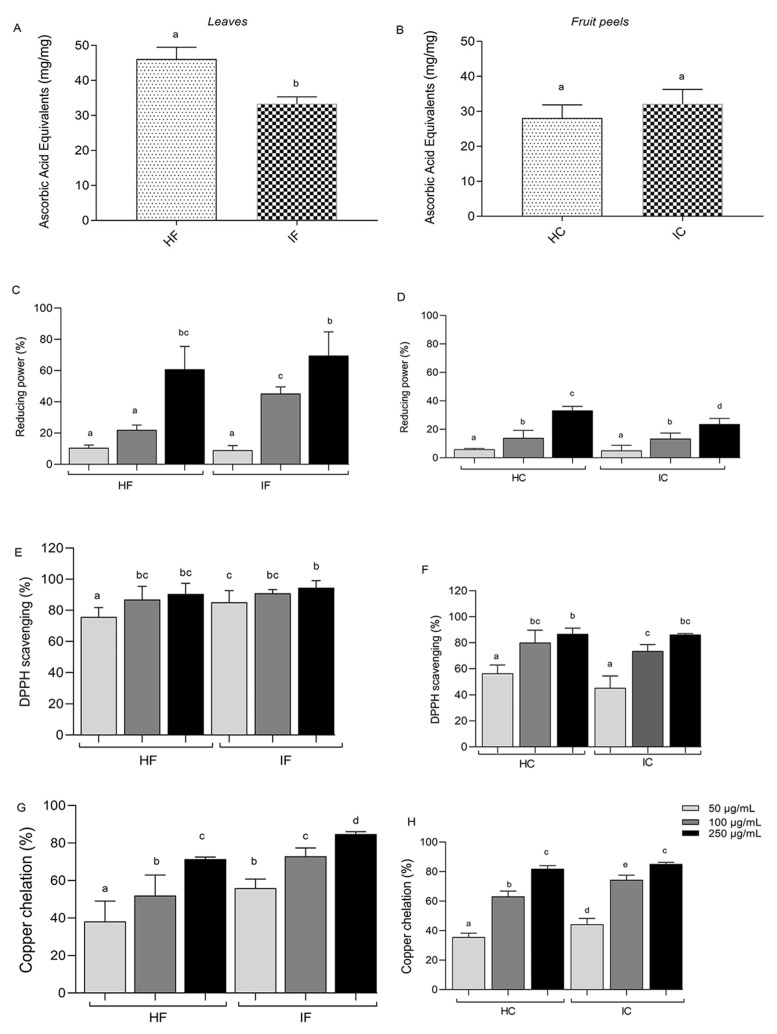
*In vitro* antioxidant capacity of *T. esculenta* extracts: (**A**) TAC in leaves, (**B**) TAC in fruit peels, (**C**) reducing power in leaves, (**D**) reducing power in fruit peels, (**E**) scavenging of DPPH radical in leaves, (**F**) scavenging of DPPH radical in fruit peels, (**G**) copper chelation in leaves, (**H**) copper chelation in fruit peels. HF corresponds to the hydroethanolic extract obtained with the leaves, IF corresponds to the infusion extract obtained with the leaves, HC corresponds to hydroethanolic extract obtained with the fruit peels, and IC corresponds to the infusion obtained with the fruit peels. Different letters (a–e) represent the statistically significant differences among treatments using Tukey test (*p* < 0.05).

The leaves and fruit peels of *T. esculenta* were submitted to hydroethanolic and infusion extractions. The antioxidant activity of the four extracts (HF—hydroethanolic leaf, IF—infusion leaves, HC—hydroethanolic peels, and IC—infusion peels) was assessed using four different antioxidant assays. The total antioxidant activity (TAC) analysis revealed that the leaf extracts exhibited a higher activity than the peel extracts (Figure 1). However, while HF was significantly higher than IF (Figure 1A), no difference was observed between the peel extracts (Figure 1B). The TAC values for the leaf extracts reached 45 mg ascorbic acid equivalent per milligram of the sample for HF and 32 mg ascorbic acid equivalent per milligram of the sample for IF. The TAC values for HC and IC were around 30 mg ascorbic acid equivalent per milligram of the sample.

The reducing capacity assay demonstrated a dose-dependent response for all samples, with the leaf extracts (HF and IF) reaching approximately 60% reducing capacity (Figure 1C) and the fruit peel extracts (HC and IC) reaching around 20% at a concentration of 250 µg/mL (Figure 1D). As shown in Figure 1E,F, all leaf and fruit peel extracts showed an interesting DPPH radical scavenging ability, with a scavenging rate of up to 80%. Only the lower concentration was statistically different among the DPPH activity observed. The copper ion chelation capacity assay indicated that both the leaf (Figure 1G) and fruit peel extracts (Figure 1H) exhibited approximately 60% activity at a concentration of 100 µg/mL. Overall, these results indicate that all four extracts (IF, HF, IC, and HC) possess an antioxidant potential in a dose-dependent manner, as demonstrated by the different antioxidant assays conducted.

The antioxidant tests used to evaluate the *in vitro* antioxidant activity, including the colorimetric tests used here, have been extensively employed to determine the capacity of compounds to counteract free radicals and to provide defense against oxidative damage [50]. However, these tests also exhibit certain limitations [51]. For instance, they often gauge a compound’s capability to counter free radicals generated within simplified chemical systems, which may not always accurately mirror the intricacies of oxidative processes taking place within the human body. This discrepancy can potentially lead to an overestimation of antioxidant activity in relation to the actual biological environment [52]. Moreover, these tests are inadequate for assessing the bioavailability and metabolization parameters of the antioxidant agents under evaluation.

Furthermore, numerous antioxidants exert their advantageous effects not solely by neutralizing free radicals, but also via intricate interactions with other molecules and cellular processes. *In vitro* tests might fall short in capturing these multifaceted effects. Lastly, in vitro antioxidant tests lack the capability to evaluate cell signaling pathways, gene expression, and other pertinent biological mechanisms that could be linked to the mode of action of the antioxidant agent under scrutiny [53]. As a result, it remains essential to supplement in vitro investigations with studies involving cultured cells and in vivo models.

### 3.2. Effect of T. esculenta Extracts on the NIH/3T3 Cell Line

**Figure 2:**
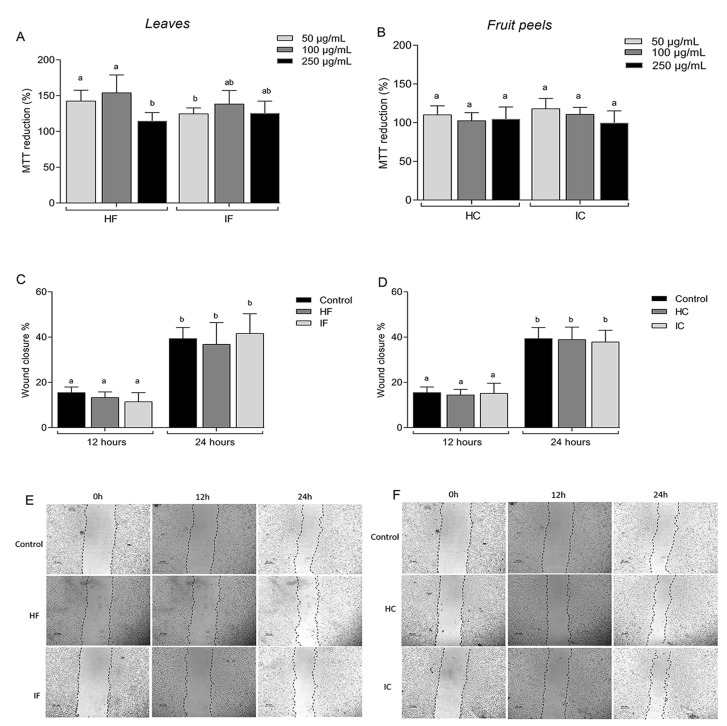
*T. esculenta* extracts’ effect on the wound healing assay. (**A**) MTT reduction capacity on NIH/3T3 cells treated for 24 h with *T. esculenta* leaf extracts. (**B**) MTT reduction capacity on NIH/3T3 cells treated for 24 h with *T. esculenta* fruit peels extracts. (**C**) Effect of *T. esculenta* leaf extracts (100 µg/mL) on the wound healing assay. (**D**) Effect of *T. esculenta* fruit peels extracts (100 µg/mL) on the wound healing assay. (**E**) Representative images of the migration assay after 0, 12, and 24 h of treatment with the HF and IF extracts. (**F**) Representative images of the migration assay after 0, 12, and 24 h of treatment with HC and IC. HF corresponds to the hydroethanolic extract obtained with the leaves, IF corresponds to the infusion extract obtained with the leaves, HC corresponds to the hydroethanolic extract obtained with the fruit peels, and IC corresponds to the infusion obtained with the fruit peels. Different letters (a,b) represent the statistically significant differences among treatments for the Tukey test (*p* < 0.05).

Taking into consideration the antioxidant potential of all *T. esculenta* extracts obtained from both the leaves and fruit peels, a parallel evaluation of their cytotoxicity was tested in different concentrations (50 µg/mL, 100 µg/mL, and 250 µg/mL) to determine whether they exerted any harmful effects on cellular viability. The results depicted in Figure 2A (leaf extracts) and Figure 2B (fruit peel extracts) reveal that the extracts did not exhibit a reduction in MTT reduction capacity. In fact, all of the observed values were higher than 100%. The next step involved investigating the potential impact of the four extracts on cell migration using a wound healing migration assay. As shown in Figure 2C–F, the treatment with extracts at a concentration of 100 µg/mL did not exhibit a significant effect on cell migration compared with the negative control after 12 h and 24 h of wound creation. These results suggest that both extracts do not have any activity in cell migration for the NIH/3T3 cell line under the evaluated conditions.

### 3.3. Effect of T. esculenta Extracts on the NIH/3T3 Fibroblast Cell Line Exposed to Oxidative Stress

**Figure 3:**
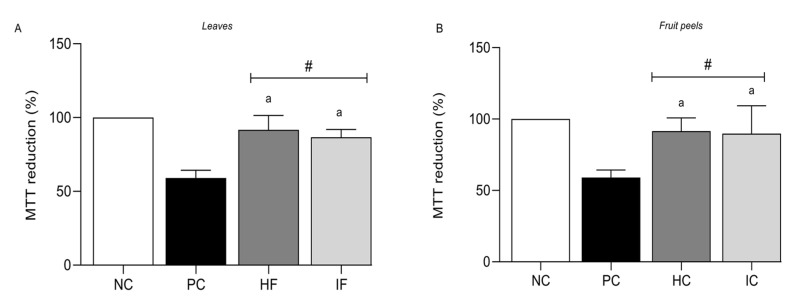
Protection effect against copper stress by *T. esculenta* extracts. (**A**) MTT reduction percentage when NIH/3T3 cell line was exposed to copper stress and was treated with leaves extracts (HF and IF). (**B**) MTT reduction percentage when the NIH/3T3 cell line was exposed to copper stress and was treated with fruit peel extracts (HC and IC). HF corresponds to the hydroethanolic extract obtained with the leaves, IF corresponds to the infusion extract obtained with the leaves, HC corresponds to the hydroethanolic extract obtained with the fruit peels, and IC corresponds to the infusion obtained with the fruit peels. The negative control (NC) corresponds to the culture medium only. The positive control (PC) represents a culture medium containing 25 µM CuSO_4_ and 1 mM ascorbate. Values are expressed as mean ± standard deviation. # indicates statistical significance compared with the positive control (*p* < 0.001). ^a^ represents the statistically significant differences among treatments for the Tukey test (*p* < 0.05).

As the *T. esculenta* extracts did not have any cytotoxic effect on the NIH/3T3 cell lines, their protective effect against the oxidative stress condition was evaluated. All of the extracts were able to protect the NIH/3T3 cell line against the oxidative stress promoted by 25 μM CuSO_4_ + 1 mM ascorbate (Figure 3). The treated cells maintained MTT reduction values of 90%, close to the control conditions (NC).

### 3.4. Effect of Leaf Infusion and Fruit Peels Hydroethanolic Extract of T. esculenta on Tenebrio molitor

**Figure 4:**
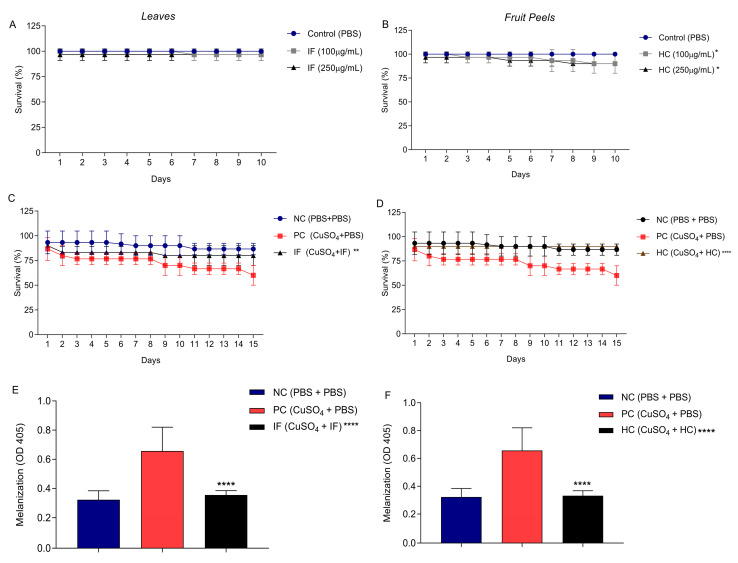
Oxidative protection from IF and HC extracts on *T. molitor* larvae. (**A**) Effect of the leaf infusion on *T. molitor* survival. (**B**) Effect of the hydroethanolic fruit peel extract on *T. molitor* survival. Larvae (*n* = 30 per group) were inoculated with concentrations of 100 and 250 µg/mL and evaluated for 10 days for survival. The control group consisted of larvae inoculated only with phosphate-buffered saline (PBS). (**C**) The effects of the leaf infusion (IF) on the survival of *T. molitor* larvae exposed to CUSO_4_. (**D**) Effects of hydroethanolic fruit peel extract (HC) on the survival of *T. molitor* larvae exposed to CUSO_4._ Survival was evaluated for 15 days. The negative control (NC) consisted of larvae that received two inoculations with PBS. The positive control (PC) consisted of larvae inoculated with 25 µM CUSO_4_ and that received the second inoculation with PBS. (**E**) Effect of the leaf infusion on the melanization of *T. molitor* larvae after 15 days of inoculation. (**F**) Effect of hydroethanolic fruit peel extract on the melanization of *T. molitor* larvae after 15 days of inoculation. Values are expressed as mean ± standard deviation. Asterisks indicate statistical difference compared with PC using Dunnett test. * corresponds to *p* < 0.05; ** corresponds to *p* < 0.01; **** corresponds to *p* < 0.001.

Larvae of Tenebrio molitor were inoculated with two different concentrations (100 µg/mL and 250 µg/mL) of IF or HC extracts of *T. esculenta* to evaluate their potential toxicity. The survival rate was monitored over a period of 10 days. Both extracts, IF and HC, did not show any toxicity with the two concentrations, reaching 100% and 90% larval survival, respectively (Figure 4A,B).

Subsequently, the IF and HC extracts were evaluated for their potential protection of *T. molitor* larvae against oxidative stress induced by CuSO_4_. Larvae survival showed a 30% decrease after CuSO_4_-induced stress (Figure 4C,D). However, when the larvae were inoculated with the IF extract at a concentration of 250 µg/mL, an impressive 80% larval survival rate was observed (Figure 4C). Similarly, the HC extract exhibited a significant protective effect, with a survival rate of 90% (Figure 4D), similar to the negative control, while the survival rate of the larvae inoculated with just PBS, after the oxidative stress, was around 50%. These results highlight the potential of both IF and HC extracts in mitigating oxidative stress and safeguarding the survival of *T. molitor* larvae.

The extracts’ ability to mitigate CuSO_4_-induced stress in the *T. molitor* larvae was also measured using the melanization level (Figure 4E,F). A higher optical density value was detected for the CuSO_4_ (0.657) stress-induced larvae when compared to the negative control (0.322). On the other hand, both IF (0.355) and HC (0.332) extracts exhibited a significant two-fold reduction in melanization compared with the CuSO_4_ stress-induced larvae, with a statistically significant difference, reinforcing their potential as protective agents against oxidative stress.

### 3.5. Detection of Biomolecules Present in the Extracts

The determination of the total phenolic compound content in the leaf infusion (IF) revealed a value of 22.598 ± 1.56 mg gallic acid equivalent (GAE)/mg, while the total flavonoid content was found to be 0.1330 ± 0.03 mg quercetin equivalent (QE)/mg. In addition, for the hydroethanolic extract of fruit peels (HC), the total phenolic compound content was measured at 19.067 ± 0.92 mg GAE/mg, indicating a slightly lower value compared with the leaf infusion. The flavonoid content was determined to be 0.0603 ± 0.0017 mg QE/mg.

**Figure 5:**
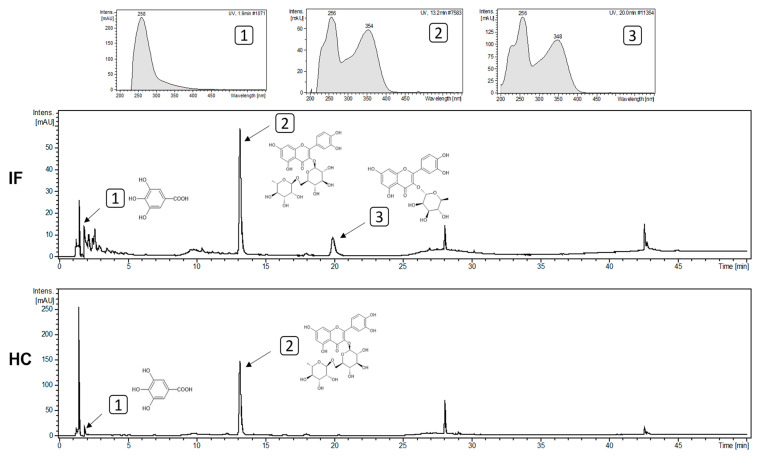
Chromatographic profile of the leaf infusion (IF) and the hydroethanolic extract of the fruit peels (HC) of *T. esculenta* obtained through the analysis with CLAE-DAD at a wavelength of 352 nm. Each number indicates a different molecule. In the chromatograms, peak 1 corresponds to gallic acid, peak 2 corresponds to rutin, and peak 3 to quercitrin. The molecule structure of the identified peaks are drawn. The *x*-axis corresponds to the retention time (min) of each peak, and the *y*-axis corresponds to the intensity of the peaks in mAU.

A CLAE-DAD analysis was conducted to gain insights into the biomolecules present in the extracts (Figure 5). In the leaf infusion (IF), three peaks were tentatively suggested based on their retention times and through a comparison with standard compounds: gallic acid (peak 1, tr = 1.9 min—phenolic acid), rutin (peak 2, tr = 13.2 min—flavonol), and quercitrin (peak 3, tr = 20 min—flavonol). In the hydroethanolic extract of fruit peels (HC), the two former peaks were also detected, while quercitrin was not. Rutin was the predominant compound in both extracts. Further analyses are required for the tentative identification of the other compounds.

## 4. Discussion

The oxidative stress in our cells play an important role in the initiation and progression of different chronic diseases such as cancer, atherosclerosis, and Alzheimer’s disease [2,54,55]. Thus, the search for antioxidant molecules that can act in the modulation of oxidative stress, regulating free radicals or the products of their oxidation, becomes an area of great relevance for the discovery of therapeutic alternatives in order to prevent and treat different pathologies [56,57]. As a result of the presence of different bioactive molecules in medicinal plants, their preparation as teas, hydroethanolic extracts, or supplements may be considered as an alternative therapeutic and nutraceutical approach [58,59,60]. Thus, the search for different antioxidant nutraceuticals, such as medicinal plants, whose use can supplement phytochemical intake and promote human health, has intensified [61,62,63]. Moreover, the recently obtained scientific data have shown that continuous pharmacological screening of different plants extracts is still an important step towards the development of new natural products, phytotherapeutics, and nutraceuticals to be used for the prevention and treatment of diseases [64].

In this study, the antioxidant potential of *T. esculenta* extracts from leaves and from fruit peels, which are typically considered as crop rejects, was evaluated. Different research groups have shown that peels and other tissues that are considered as rejects or waste can be an excellent source for the identification of new bioactive molecules, as well as for the identification of new activities from known biomolecules [65,66,67,68]. Taking this into consideration, we also investigated the antioxidant activities of fruit peels, recognizing their potential as a valuable resource for antioxidant compounds. The biochemistry data of the hydroethanolic extracts and the infusion preparations showed that these extracts might be able to act as free radical scavengers, electron donors, and metal chelators, which can be associated with the different bioactive molecules present in these extracts. Bajpai et al. [69], working with extracts, observed that the combination of different biomolecules may work in synergy with the antioxidant activity.

The DPPH assay showed that HF and IF had approximately 80% activity at the lowest evaluated concentration (50 µg/mL), and HC and IC had 50% of DPPH radical sequestration in a 50 µg/mL concentration and 90% at 250 µg/mL. Fraga et al. [21] reported the antioxidant capacity of the ethanolic extract of *T. esculenta* fruit peels using six different antioxidant assays, achieving an 80% DPPH radical scavenging with an 80-fold concentration, which was used in the present study. Jantapaso and Mittraparp-Arthorn [70], evaluating aqueous extracts of fruit peels of *Nephelium lappaceum* L., another species of Sapindaceae, described an EC50 of 494.25 µg/mL for the DPPH assay, a value much higher than observed with *T. esculenta*. In addition [71], the EC50 values observed for the leaf and fruit peel extracts of *Nephelium lappaceum* concerning DPPH were 320 µg/mL and 35 µg/mL, respectively. These differences might be associated with the specific extraction methods, solvents used, and time of extraction [64,72]. The data presented here show that the leaf extract of *T. esculenta* was six times more effective at scavenging 50% of the DPPH radical when compared with *Nephelium lappaceum*, reinforcing the potential of the former as an antioxidant. Despite this, it should be noted that spectrophotometry assays, due to their nature, provide a suggestive measure of the antioxidant capacity of the extracts, but do not accurately reflect the antioxidant mechanism of the compounds when assessing the antioxidant capacity of the plant extracts, hence the importance of using different assays and combining them with experiments on biological systems such as cells and animals in order to obtain a more complex result [34,73].

The effects of *T. esculenta* extracts on the NIH/3T3 fibroblast cell line showed no cytotoxicity effects, which is an important characteristic for natural products in order to be considered for safe and viable application by the population [33,74]. The findings from our study are consistent with previous research conducted by [21], who also observed antioxidant effects in different cell lines. Additionally, the study by [75] with *Litchi chinensis* Sonn (Sapindaceae) supported our results. However, the authors of [71] observed a cytotoxicity effect from the extracts obtained with *Nephelium lappaceum* leaves and fruit peels on human skin fibroblasts.

Prolonged exposure to copper sulfate can lead to mitochondrial dysfunction, induction of apoptosis, and reduced activity of the antioxidant enzymes [76,77,78,79]. Furthermore, ascorbate can potentiate the action of copper in free radical formation by rapidly reacting with the oxidized form of copper, reducing it to Cu^+^, thereby providing more substrate for hydroxyl radical formation [2,80,81]. In addition, the excess, as well as the accumulation of copper in tissue can lead to severe neurological diseases such as Parkinson’s, Wilson’s disease, and amyotrophic lateral sclerosis; furthermore, high levels of copper can also influence the development of atherosclerosis and hypertension, diabetes mellitus, and obesity [82,83,84,85]. The relationship of copper with the formation and progression of diseases is mainly associated with its strong role in the production of reactive species such as H_2_O_2_ and OH; these oxidants, in turn, exert harmful effects by reacting strongly with proteins, lipids that make up membranes, DNA, and other cellular components, causing chemical modifications and loss of function, and ultimately compromising the functioning of organs and contributing to the development of pathologies [83,86]. In addition, it disrupts the signaling pathways that regulate important cellular processes such as apoptosis, autophagy, and glycolysis and stimulates inflammatory signaling, which are factors typical of several diseases [87,88,89]. Besides the high dose-dependent activity of cooper chelating obtained in vitro (Figure 1), the antioxidant capacity of *T. esculenta* extracts was also confirmed using the CuSO_4_-stressed NIH/3T3 cell line and *Tenebrio molitor* as in vivo models. The four extracts from *T. esculenta* were able to protect the NIH/3T3 cell line against oxidative stress at almost the same level as that of the negative control. This protective effect against oxidative damage can be attributed to the presence of antioxidant biomolecules that may inactive or scavenge the formation of reactive oxygen species [58,90]. Different research with other Sapindaceae species (*Paullinia cupana* Kunth, *Nephelium lappaceum,* and *Litchi chinensis*) showed antioxidant protection against radiation or hydrogen peroxide [75,91,92]. In this study, we first show protection against copper.

The utilization of insects as an invertebrate experimental model for in vivo studies has gained increasing recognition due to their varied advantages, such as life cycle and easy maintenance in laboratory conditions and high reproducibility [93,94]. *Tenebrio molitor*, commonly known as mealworm, has recently been used as a model organism for studying pathogenic fungal infections and for screening of antifungal drugs [95,96,97]. *T. molitor.* Furthermore, *T. molitor* is currently being utilized for assessing the in vivo antioxidant properties of plant extracts and as a toxicity model. Silva et al. [44] employed *T. molitor* larvae to analyze the effects of *Buchenavia tetraphylla* against oxidative stress induced by inactivated *E. coli*. In addition, Azevedo et al. [98] measured the protective effects of *Peumus boldus* and *Matricaria chamomilla* teas on *T. molitor* larvae, after the induction of oxidative stress by LPS. In their recent study, Brai et al. [99] analyzed the toxicity of drugs with different pharmacological applications (antibiotics, neuroleptics, analgesics, antihistamines, and anti-inflammatories) in *T. molitor* and observed that the insect could be used as an efficient alternative for evaluating the acute toxicity of compounds.

The results observed for the assays with IF and HC against CuSO_4_-induced oxidative damage in *T. molitor* corroborated the antioxidant activity, as evidenced through both the biochemistry and NIH/3T3 cell line. The massive influence of CuSO_4_ exposure on the larval development and survival rate of *Galleria mellonella* due to oxidative stress has already been described. For example, it has been verified that CuSO_4_ is a source of oxidative stress for invertebrate models [100,101]. Tuncsoy et al. [102] also observed a modulation on the antioxidant enzyme activity for *G. mellonella* larvae exposed to copper oxide nanoparticles. Here, an extract protection on *T. molitor* larvae previously submitted to CuSO_4_ exposure was shown, revealing the potential of *T. esculenta* extracts in mitigating oxidative damage promoted by copper oxidative stress.

The CLAE-DAD analysis revealed the presence of flavonoids and phenolic acids in the four extracts. The suggested identification of the peaks corresponded to specific biomolecules, including gallic acid (a phenolic acid), rutin (a flavonol glycoside), and quercitrin (a flavonol glycoside). Flavonoids are well-known for their potent antioxidant properties. They can scavenge free radicals by donating hydrogen atoms and electrons, thereby stabilizing the radicals and preventing oxidative damage. Additionally, flavonoids can chelate metal ions such as copper, thus inhibiting the formation of free radicals and reducing their reactivity [103,104,105]. Numerous studies have demonstrated the antioxidant and anti-inflammatory effects of quercitrin, rutin, and gallic acid both in vitro and in vivo [106,107,108,109,110]. De Souza et al. [111] identified the presence of gallic acid, along with other compounds such as quinic acid, catechin, epicatechin, chlorogenic acid, caffeic acid, *p*-coumaric acid, ferulic acid, and quercetin in the *T. esculenta* extracts using HS-SPME/GC-MS and LC-MS/MS methods. Fraga et al. [21] also identified rutin in *T. esculenta* fruit peel extracts. Similarly, the fruit peel from *Dimocarpus longan* (Sapindaceae) showed the presence of gallic acid, chlorogenic acid, corilagin, ferulic acid, ellagic acid, *p*-coumaric acid, quercetin, and kaempferol [112]. These data showed that some biomolecules can be markers for this family, and reinforced the potential of *T. esculenta* leaves and fruit peels as a natural source of bioactive molecules with pharmacological properties.

Our results provide evidence of the pharmacological potential of the infusion of *T. esculenta* leaves, a preparation used in folk medicine, and it allowed for detecting phenolic components that may be associated with the activities presented here. Moreover, the use of fruit peels could be of economic importance as a reject product, and could stimulate the use of a potential waste resources in innovative biotechnological applications, such as the development of natural food supplements with different antioxidant effects that can help protect against and combat many diseases. However, more studies are needed to better characterize the bioactive compounds present in the extracts, with a view to identify and quantify them using additional phytochemical analysis techniques. In addition, as these are raw extracts with a complex composition, their toxicity and therapeutic actions need to be further evaluated to ensure efficacy and safety.

## 5. Conclusions

The present study provides evidence that extracts obtained from the leaves and fruit peels of *T. esculenta* exhibit an important in vitro and in vivo antioxidant potential. Moreover, the leaf infusion (used medicinally) and the hydroethanolic extract of the fruit peels (a biological reject) showed the ability to protect *T. molitor* larvae against oxidative damage induced by CuSO_4_ exposure. Furthermore, the phytochemical analysis revealed the presence of bioactive molecules such as gallic acid, rutin, quercitrin, and others not identified here. All of these bioactive molecules are known to have a potential pharmacological activity. Nonetheless, our results highlight the potential of *T. esculenta* extracts as a valuable resource for exploring the application of natural products in nutraceutical and pharmaceutical fields. However, further studies on the effects of the consumption of *T. esculenta* extracts as a bioactive food supplement should be conducted.

## Data Availability

The data obtained in this study are available from the corresponding author upon request.
